# Comparison and Analysis on Mechanical Property and Machinability about Polyetheretherketone and Carbon-Fibers Reinforced Polyetheretherketone

**DOI:** 10.3390/ma8074118

**Published:** 2015-07-07

**Authors:** Shijun Ji, Changrui Sun, Ji Zhao, Fusheng Liang

**Affiliations:** School of Mechanical Science and Engineering, Jilin University, Changchun 130025, China; E-Mails: jishijun@jlu.edu.cn (S.J.); suncr14@mails.jlu.edu.cn (C.S.); liangfusheng@mails.jlu.edu.cn (F.L.)

**Keywords:** polyetheretherketone, 30% carbon-fiber reinforced polyetheretherketone, nano-indentation, single-point diamond turning

## Abstract

The aim of this paper is to compare the mechanical property and machinability of Polyetheretherketone (PEEK) and 30 wt% carbon-fibers reinforced Polyetheretherketone (PEEK CF 30). The method of nano-indentation is used to investigate the microscopic mechanical property. The evolution of load with displacement, Young’s modulus curves and hardness curves are analyzed. The results illustrate that the load-displacement curves of PEEK present better uniformity, and the variation of Young’s modulus and hardness of PEEK both change smaller at the experimental depth. The machinability between PEEK and PEEK CF 30 are also compared by the method of single-point diamond turning (SPDT), and the peak-to-valley value (PV) and surface roughness (Ra) are obtained to evaluate machinability of the materials after machining. The machining results show that PEEK has smaller PV and Ra, which means PEEK has superior machinability.

## 1. Introduction

Polyetheretherketone (PEEK) materials belong to a group of high-performance thermoplastic polymers [1]. Among various polymers, polyetheretherketone (PEEK) is a representative increasingly used in tribological components because of its excellent performance such as high mechanical properties, good wear-resistance and heat-resistance, and excellent chemical resistance, *etc.* [2,3,4,5,6]. PEEK has also many applications in engineering and medicine because of its high strength and high melting point relative to other polymers, as well as its resistance to chemical and biological action [7,8,9].

Carbon fiber (CF) is a widely-used filler because of its excellent performance such as high strength and modulus, low thermal expansion, good creep-resistance and corrosion-resistance, *etc.* [10,11,12,13,14,15]. Carbon fibers are invariably used in advanced composites for special applications such as in automobile and aviation industries [16,17]. Carbon Fiber Reinforced Plastics (CFRPs) are gaining wide acceptance in the aeronautic sector, for the excellent strength to weight ratio offered by the laminates. Among all polymers used as matrixes, polyetheretherkethone (PEEK) is an advanced thermoplastic resin that provides higher toughness and better resistance to abrasive wear with respect to thermoset resins [18,19]. CF/PEEK laminates also show a useful increasement in service temperature since the thermoplastic matrix offers excellent mechanical and chemical resistance and keeps these properties up to remarkably high service temperature [1,18]. Molazemhosseini A. [20] found that the local nanomechanical properties of the composites highly depend on the type of the existing phase which is indented and the incorporation of short carbon fibers into neat PEEK results in a remarkable enhancement in the reduced elastic modulus and hardness of the material (143% increase in reduced elastic modulus).

In nano-indentation, the probe and loads are very small, so as to produce indentations a few micrometers to a few hundred nanometers in size [21]. Therefore, nano-indentation is commonly used for the study of mechanical properties of materials on the nanoscale [22]. The single-point diamond turning process is capable of producing components with micrometer to submicrometer form accuracy and surface roughness in the nanometer range [23]. Thus, it can be used to measure the machinability of materials in micro scale.

In this paper, the microscopic mechanical property and machinability are compared between PEEK and PEEK CF 30 to better understand their characteristic. The focus of the paper is the differences between the microscopic mechanical property and machinability for pure PEEK and 30% of carbon fibers reinforced PEEK; the study can make a better spread of utilization for the materials. Nano-indentation is used to investigate the mechanical property, and the single-point diamond turning (SPDT) is chosen to study the machinability.

## 2. Materials and Experimental Procedure

**Figure 1:**
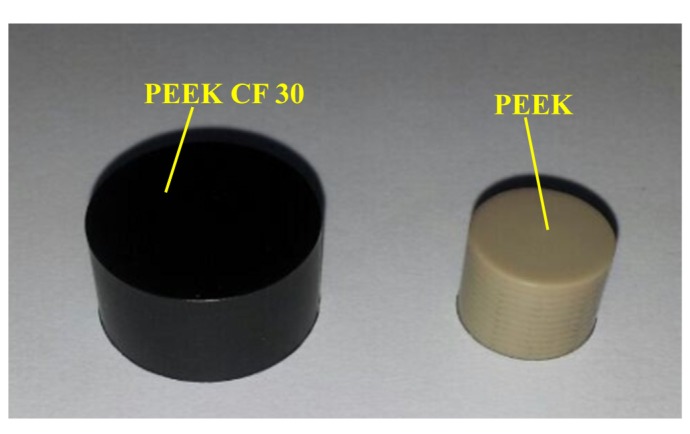
Composite materials.

The experiments are carried out with several cylindrical samples which are produced by the method of extrusion. The brief process of manufacturing the cylindrical samples is introduced below. The powders of PEEK and carbon fibers in a specified proportion were mixed mechanically for 10 min. A mold was prepared to hold the mixture. Then, the mold was heated to 360 °C at the rate of 10 °C/min, and the pressure was kept at 3 MPa; after 10 min, the mixtures were cooled below 100 °C. Finally, the diameter of 10 mm for PEEK and the diameter of 16 mm for PEEK CF 30 are obtained. The tensile strength for PEEK and PEEK CF 30 are about 94 MPa and 212 MPa at the experimental temperature, respectively. The experiments performed on a cross section of the samples, and the samples can be seen in Figure 1.

Nano-indentation is a powerful and advanced way to measure mechanical properties such as Young’s modulus and hardness of various materials. This method has been widely used to study the mechanical properties of polymers and nano-composites [24,25,26,27,28,29].

**Figure 2:**
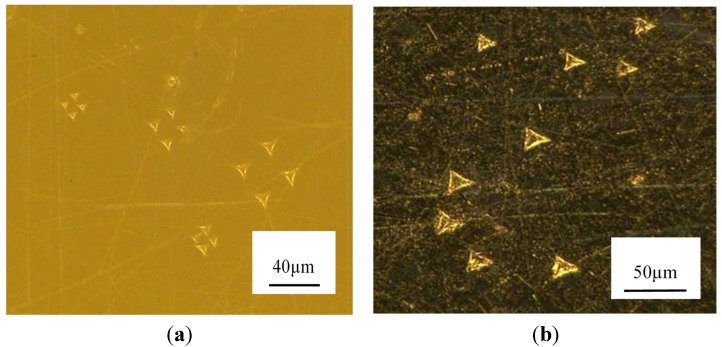
Indentation points (**a**) Polyetheretherketone (PEEK); (**b**) 30 wt% carbon-fibers reinforced Polyetheretherketone (PEEK CF 30).

In order to compare the mechanical property between PEEK and PEEK CF 30 from a micro view, Agilent G200 is used to perform the nano-indentation experiments at room temperature. The main parameters of Agilent G200 can be seen in Table 1. An indenter with triangular pyramid tip is adopted and the apex angle for the indenter is 120° and the diameter of the indenter is about 1.2 mm. To compare the property of the materials at different depth, three sets of the indentation depth are designed, which are 4 μm, 6 μm, 8 μm, respectively. To ensure that the data of the experiment is reliable, four points in different site on the samples are conducted during the tests for each indentation depth. The indentations were conducted in the velocity-control mode with the surface approach velocity being 10 nm/s. When approaching the designed depth, 2 s peak hold time is controlled. The morphologies of the indentation can be obtained in Figure 2.

**Table 1:** Main parameters of nano indenter G200.

Technical Index	Standard XP Indentation Head
Maximum indentation depth	>500 μm
Total indenter travel	1.5 mm
Displacement resolution	<0.01 nm
Maximum load (standard)	500 mN
Load resolution	50 nN
Contact force	<1.0 μN

**Figure 3:**
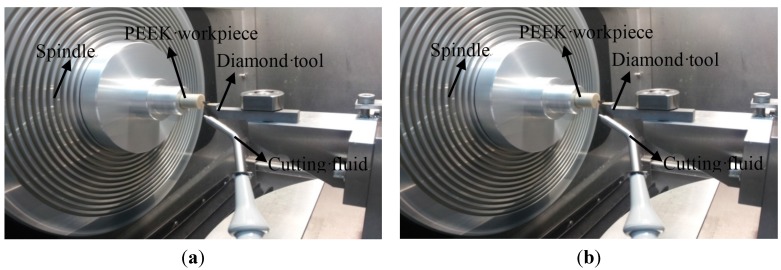
Main sections of the cutting process (**a**) PEEK; (**b**) PEEK CF 30.

The technology of single-point diamond turning (SPDT) is used to turn the prepared samples to measure the machinability for PEEK and PEEK CF 30. The tests were performed in ultra-precision lathe NANOFORM 250 and the workpieces are fixed on the spindle, which are presented in Figure 3.

Due to two planes of the cylinder being machined, X-axis and Z-axis are enough in the cutting process. Considering the characteristic of the workpieces, parameters of the machining process are set as F = 1.0 mm/min (feed rate), S = 1500 rpm (spindle speed). The parameters of the adopted cutting tool are 120° included angle, 10° clearance angle, 0.5 mm nose radius and 0° rake angle. To meet the demand of the turning, the cutting fluid is used.

**Figure 4:**
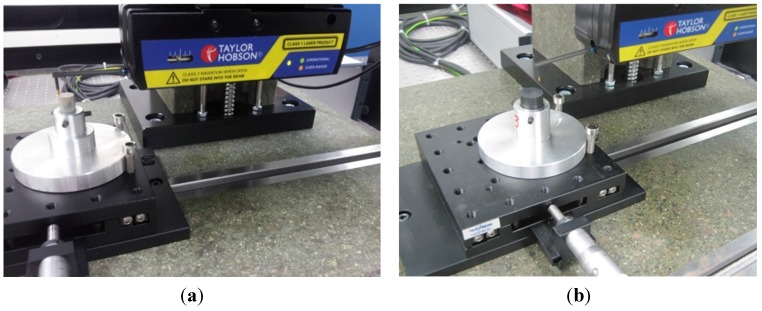
Measuring process (**a**) PEEK; (**b**) PEEK CF 30.

The form accuracy and surface roughness of the machined materials was tested using TAYLOR HOBSON which measures the materials through the mechanism of contact probe. The measuring process is given in Figure 4.

## 3. Results and Discussion

### 3.1. Results Analysis for Mechanical Property

**Figure 5:**
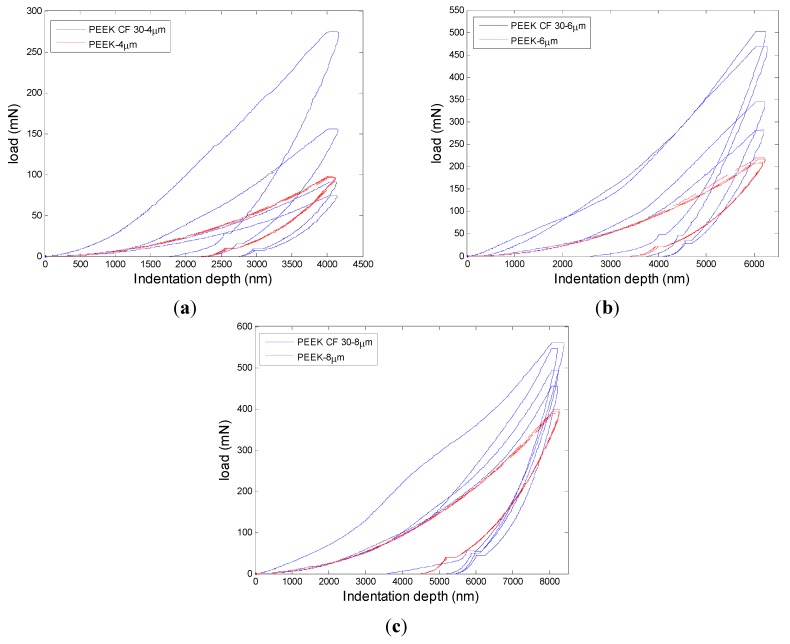
Load as function of indentation depths for both materials (**a**) 4 µm; (**b**) 6 µm; (**c**) 8 µm.

PEEK just owns one material while PEEK CF 30 has two kinds of materials. The components of the materials may have great influence on the mechanical properties. During the indentation, load and indentation depth can be continuously recorded, leading to curves of load in function of displacement, as shown in Figure 5, which will help better understand the mechanical properties of the materials. The nano-indentation data of the three tests at the max displacement are shown in Table 2.

**Table 2:** Nano-indentation data at max load.

Nano-Indentation Depth	PEEK		PEEK CF 30	
Designed depth (nm)	Average max load (mN)	Average max depth (nm)	Average max load (mN)	Average max depth (nm)
4000	96.218	4107.389	147.523	4146.987
6000	211.964	6203.548	396.228	6228.519
8000	390.710	8256.962	451.449	8223.992

**Figure 6:**
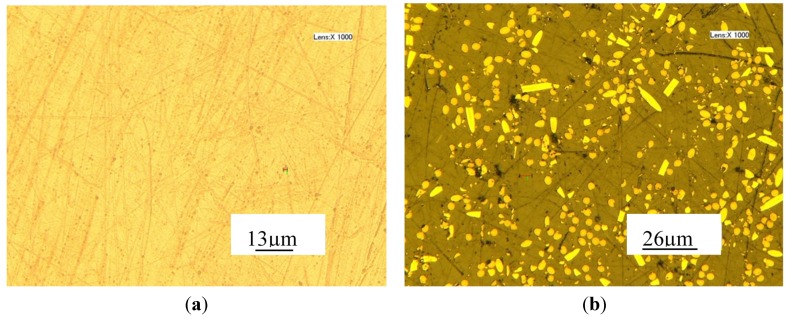
Material surface magnified a thousand times (**a**) PEEK; (**b**) PEEK CF 30.

From the three groups of curves shown in Figure 5, it can be seen that the fluctuation of PEEK CF 30 materials is significant. For the same depth, the variation of loads changes a lot for PEEK CF 30. With the increase of the indentation depth, the trend of the mean maximum load for PEEK and PEEK CF 30 materials both becomes bigger. It also can be seen that the coincidence of the curves in the same depth for PEEK is very good. However, the curves of PEEK CF 30 change a lot, even at the same depth. The variation trend of the curves may contribute to the components of the materials. Since the PEEK CF 30 materials are composed of two kinds of materials, the inhomogeneous distribution of the carbon-fiber reinforcements is the main reason leading the phenomenon, which can be seen from Figure 6.

From Figure 5a, it can be seen that the peak load of PEEK CF 30 even smaller than that of PEEK, which may be caused by the defects of the materials. It can be seen from Figure 6 that the surface for pure PEEK is good. However, the distribution of carbon fibers is inconsistent and the size of carbon fibers is different. Further, there are some black spots which are defects on PEEK CF 30 surface and the size of the defects can significantly affect the nano-indentation results. Thus, if the nano-indentation points are just carried out on the defective area, the smaller load may be caused for PEEK CF 30 materials. But from Table 2, it can be observed that the mean max load of PEEK CF 30 is much larger than that of PEEK at similar depth. The maximum load grows with the increase of nano-indentation depth. However, it is not a linear increase, because plastic deformation occurs, which can be seen from loading-unloading curves. During the 2 s peak hold time, the displacement at different nano-indentation depth keeps increasing, which means creep deformation occurs.

**Figure 7:**
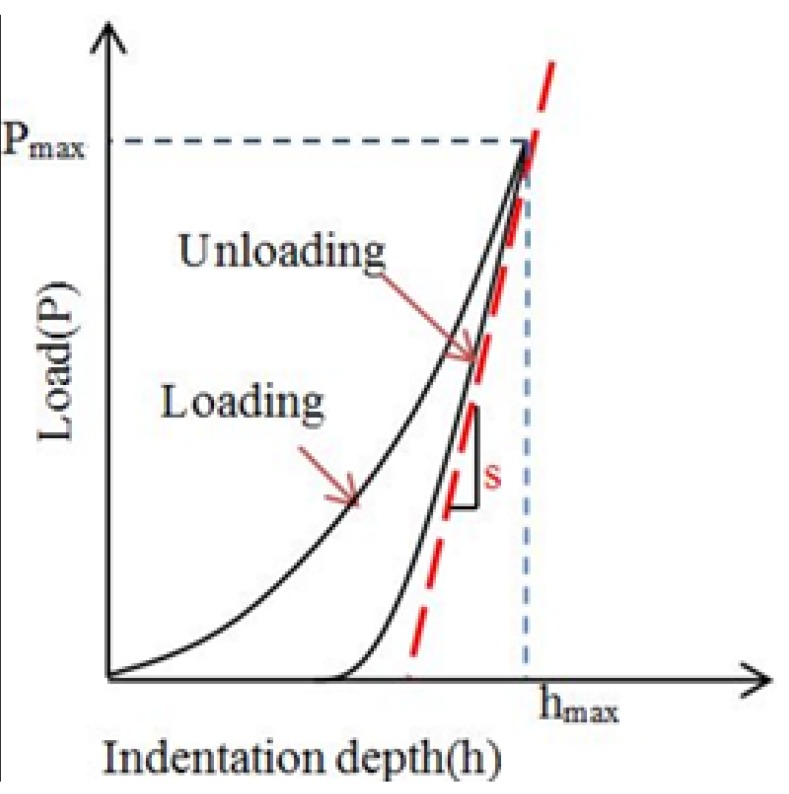
Typical loading-unloading curve of a nano-indentation test.

The reduced elastic modulus was calculated by the Oliver and Pharr method, which the loading-unloading curve was also used such as that illustrated in Figure 7 [30]. Taking account of the stiffness of the indenter being much higher than the indented materials, the hardness is defined as:

(1) $$H=\frac{P_{\max}}{A_{c}}$$

where *P_max_* is the peak force of the indentation and *A_c_* is the contact area at the maximum force.

The slope at the beginning of the unloading curve is defined as the contact stiffness *S*, which is given by the following equation:

(2) $$S=\frac{dP}{dh}$$

where *P* is the load exerted on the materials and *h* is the indentation depth. *S* can also be given by a power law:

(3) $$S=\frac{2\beta }{\sqrt{\pi }}\times {\left(\frac{1}{E_{r}}\right)}^{-1}\times \sqrt{A_{c}}$$

where β is a parameter which depends on the geometry of the indenter, *E_r_* is the reduced modulus which considers the deformation of the specimen and the indenter. The elastic modulus, *E*, of the indented specimen can be calculated by the followed equation:

(4) $$\frac{1}{E_{r}}=\frac{1-v^{2}}{E}+\frac{1-v_{i}^{2}}{E_{i}}$$

where *E_i_* is the elastic modulus of the indenter and *v_i_* is the Poisson’s ratio of the indenter, *v* is the Poisson’s ratio of the text sample.

**Figure 8:**
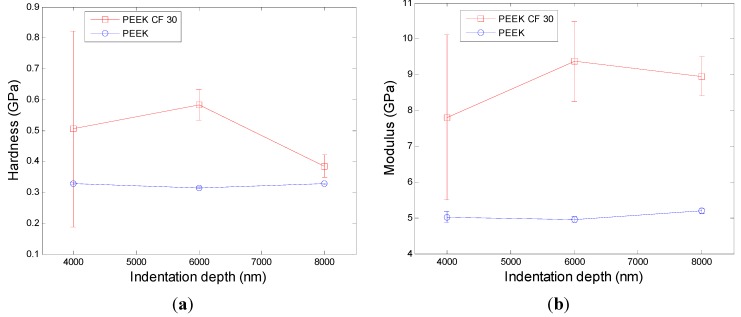
Hardness and Modulus at max load in different depth. (**a**) the hardness and (**b**) the modulus for PEEK

Based on the theory mentioned above, the Agilent G200 can automatically get the data of hardness and modulus at different depth. Then the hardness and the modulus for PEEK and its composition at max load in different depth are illustrated in Figure 8.

It can be seen from Figure 8 that the hardness and modulus for PEEK show good consistency and for each depth the variation is small. For PEEK CF 30, the fluctuation of hardness and modulus is huge, but the variation changes smaller with the nano-indentation depth becomes deeper, which may be caused by the influence of the defects of materials becoming smaller. The defects were caused by the dispersion of carbon fibers being inconsistent, which can be seen from Figure 6. At different depths, both the hardness and modulus for PEEK CF 30 are larger than that for PEEK, which means that PEEK CF 30 has superior mechanical properties.

### 3.2. Results Analysis for Machinability of the Materials

Since roughness can largely affect the performance of a mechanical component, it was chosen to measure the machinability of the materials. Moreover, Ra is the most widely-used roughness parameter, which means arithmetic average roughness. Thus, Ra is used to characterize the surface roughness.

**Figure 9:**
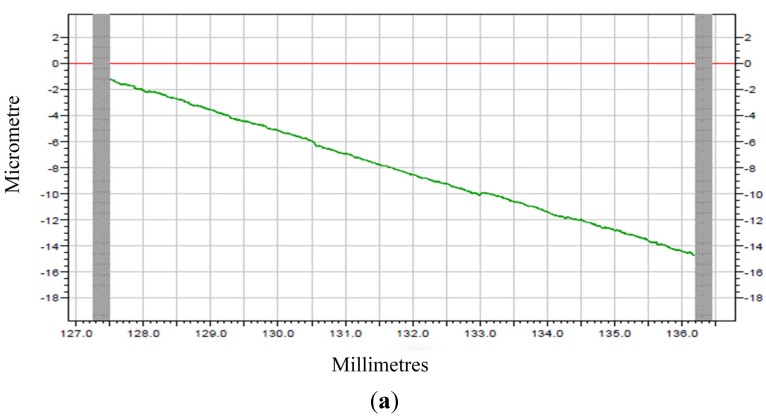

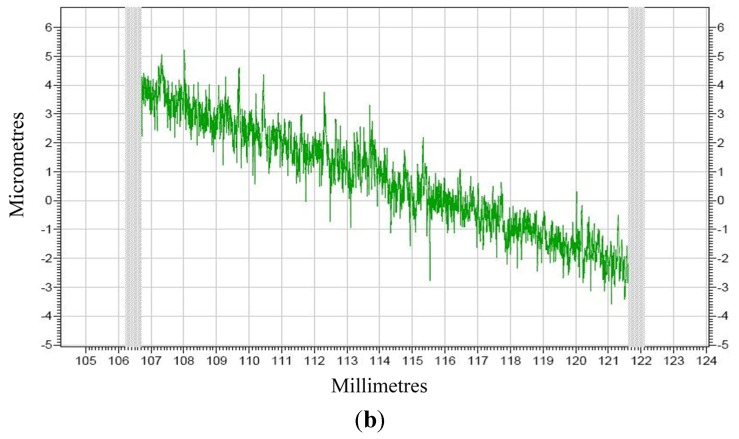
Raw profiles of machined surface (**a**) PEEK; (**b**) PEEK CF 30.

**Figure 10:**
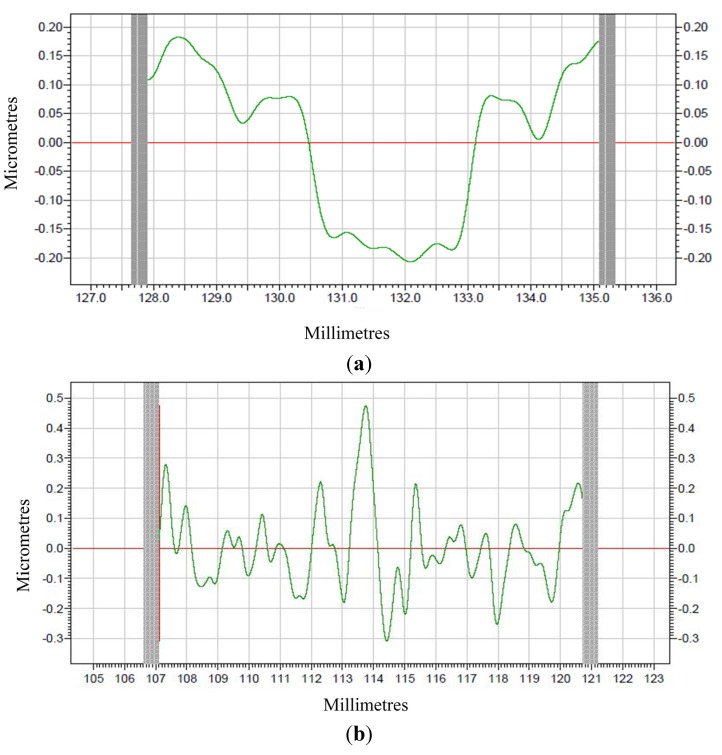
The form accuracy of machined surface (**a**) PEEK; (**b**) PEEK CF 30.

**Figure 11:**
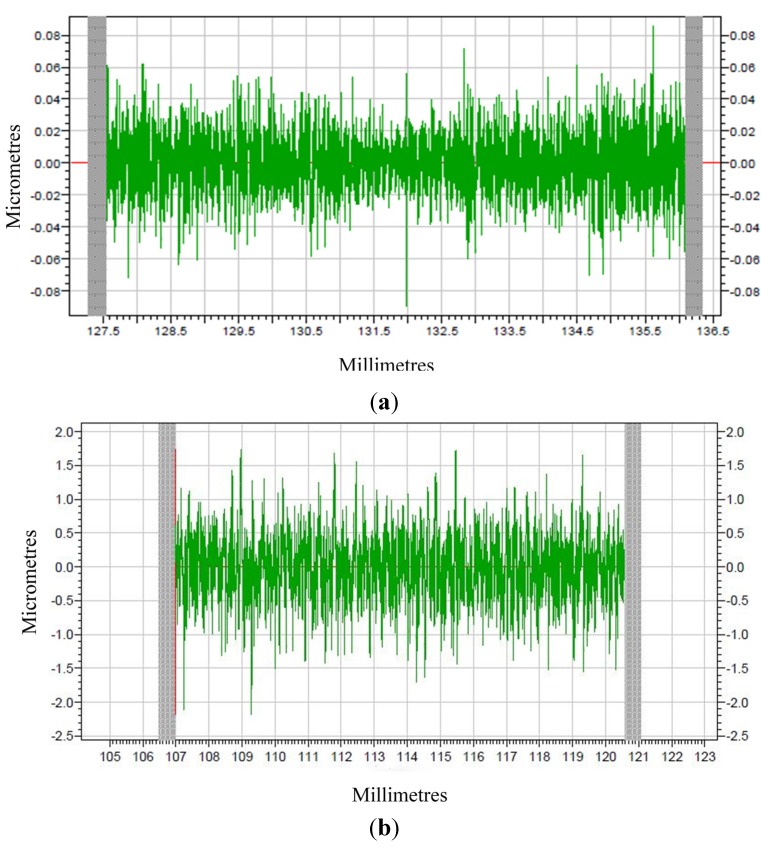
The surface roughness of machined surface (**a**) PEEK; (**b**) PEEK CF 30.

Raw profiles are gotten after machining which are indicated in Figure 9. The modified profiles for corresponding raw profiles are given in Figure 10 and Figure 11, which are form accuracy and surface roughness, respectively. The meaning of the ordinate for the measuring results is the error (unit: μm), and meanwhile the abscissa represents the measuring distance (unit: mm).

The peak-to-valley value (PV) is used to evaluate the form accuracy. The PV value of machined PEEK is shown in Figure 10a, and the PV value of machined PEEK CF 30 is shown Figure 10b, respectively. Figure 10a shows that the PV value is about 0.38 μm for machined PEEK, while about 0.78 μm for machined PEEK CF 30 can be obtained from Figure 10b. It is evident that PEEK materials provide better form accuracy with respect to PEEK CF 30 materials.

It can be observed from Figure 11 that the machined surface roughness (Ra) for PEEK materials is 0.0134 μm and for PEEK CF 30 materials is 0.3555 μm, which means PEEK materials present superior surface roughness in comparison to PEEK CF 30 materials. In a word, it can be concluded that the PEEK materials have better machinability than PEEK CF 30 materials.

### 3.3. Discussions

From the analysis above, it can be concluded that the mechanical property of materials has a great influence on machinability. The results of nano-indentation experiment can get the load with indentation depths curves, hardness and modulus, which can infer the distribution and defect of the materials. In the nano-indentation experiment, the large fluctuation of load with indentation depths curves shown in Figure 5 and the huge variation of the standard deviation of hardness and modulus for PEEK CF 30 materials shown in Figure 8 may be caused by inconsistent distribution of carbon fiber, which can be proved by Figure 6. Moreover, this phenomenon will lead to a poor machinability for PEEK CF 30 materials, which is demonstrated by SPDT experiment. However, because of the homogeneity for PEEK materials, which can be seen from Figure 6, it can be inferred that the force of each cutting point is relatively stable during the machining process, which results in a lower PV value and Ra, as proved by SPDT experiment.

## 4. Conclusions

Mechanical property and machinability for PEEK materials and PEEK CF 30 materials are investigated. The evolutions of load with displacement are obtained to analyze the mechanical property, and form accuracy and surface roughness are used to evaluate the machinability of the materials. This investigation leads to the following conclusions:

(1) The load-displacement curves of pure PEEK show superior uniformity at different nano-indentation depth, but the curves of PEEK CF 30 show poor repeatability and consistency.

(2) The dates of form accuracy gained form SPDT experiments illustrate that PEEK materials have smaller PV values in comparison to PEEK CF 30 materials after cutting, and the results of surface roughness about two machined surfaces show that PEEK materials has smaller Ra value. This means that PEEK has better machinability.

(3) Owing to the homogeneity for PEEK materials, the force of each cutting points presents good consistency, and the deformation and fracture property of the removal materials show little discrepancy during the removal of the materials, which leads to a lower PV value and Ra. However, poor consistency of PEEK CF30 materials results in large fluctuation of the cutting force and a big change of the deformation and fracture property for each cutting points, which leads to a higher PV value and Ra. So the machinability for PEEK CF 30 materials is poor.

(4) Based on the investigation of nano-indentation experiments, the distribution characteristics of the materials can be studied, which will help to predict and infer the machinability of the materials.
